# Yeast Ribonucleotide Reductase Is a Direct Target of the Proteasome and Provides Hyper Resistance to the Carcinogen 4-NQO

**DOI:** 10.3390/jof9030351

**Published:** 2023-03-14

**Authors:** Daria S. Spasskaya, Kirill A. Kulagin, Evgenia N. Grineva, Pamila J. Osipova, Svetlana V. Poddubko, Julia A. Bubis, Elizaveta M. Kazakova, Tomiris T. Kusainova, Vladimir A. Gorshkov, Frank Kjeldsen, Vadim L. Karpov, Irina A. Tarasova, Dmitry S. Karpov

**Affiliations:** 1 Engelhardt Institute of Molecular Biology, Russian Academy of Sciences, 119991 Moscow, Russia; 2 Center for Precision Genome Editing and Genetic Technologies for Biomedicine, Engelhardt Institute of Molecular Biology, Russian Academy of Sciences, 119991 Moscow, Russia; 3 Institute of Medical and Biological Problems, Russian Academy of Sciences, 123007 Moscow, Russia; 4 V.L. Talrose Institute for Energy Problems of Chemical Physics, N.N. Semenov Federal Research Center of Chemical Physics, Russian Academy of Sciences, 119334 Moscow, Russia; 5 Department of Biochemistry and Molecular Biology, University of Southern Denmark, 5230 Odense M, Denmark

**Keywords:** shotgun proteomics, 4-NQO, ribonucleotide reductase, *S. cerevisiae*, proteasome

## Abstract

Various external and internal factors damaging DNA constantly disrupt the stability of the genome. Cells use numerous dedicated DNA repair systems to detect damage and restore genomic integrity in a timely manner. Ribonucleotide reductase (RNR) is a key enzyme providing dNTPs for DNA repair. Molecular mechanisms of indirect regulation of yeast RNR activity are well understood, whereas little is known about its direct regulation. The study was aimed at elucidation of the proteasome-dependent mechanism of direct regulation of RNR subunits in *Saccharomyces cerevisiae*. Proteome analysis followed by Western blot, RT-PCR, and yeast plating analysis showed that upregulation of RNR by proteasome deregulation is associated with yeast hyper resistance to 4-nitroquinoline-1-oxide (4-NQO), a UV-mimetic DNA-damaging drug used in animal models to study oncogenesis. Inhibition of RNR or deletion of RNR regulatory proteins reverses the phenotype of yeast hyper resistance to 4-NQO. We have shown for the first time that the yeast Rnr1 subunit is a substrate of the proteasome, which suggests a common mechanism of RNR regulation in yeast and mammals.

## 1. Introduction

Cells are constantly exposed to external and internal factors that damage DNA and threaten genome stability. It is estimated that there are 10^4^–10^5^ DNA damages per human cell per day [1]. Dysfunction of DNA repair systems or excessive damage can lead to the transformation of DNA damage into mutations, which contribute significantly to the initiation and progression of various pathologies, such as neurodegenerative diseases [2], cancer [3], and aging [4]. Chemical mutagens in animal models are widely used to study the molecular mechanisms of carcinogenesis and the action of DNA repair systems, and to help elucidate targets of DNA repair-related cancer therapy [5].

4-NQO is considered a cancerogenic drug and is widely used in animal models to study the process of malignant cell transformation [6,7]. 4-NQO has two modes of action leading to the activation of the corresponding molecular defense mechanisms. First, it covalently modifies DNA bases to form bulk DNA adducts, which are subsequently recognized and removed in the nucleotide excision repair pathway [8]. Second, mitochondrial enzymes catalyze the conversion of 4-NQO to 4-hydroxyaminoquinoline-1-oxide, accompanied by the formation of reactive oxygen species (ROS), which oxidize guanosine in DNA to 8-hydroxideoxyguanosine and lead to the activation of antioxidant defense systems such as the glutathione system [9]. Moreover, 4-NQO-induced oxidation of RNA nucleobases causes induction of ribosome quality control and activation of the ubiquitin-proteasome system (UPS) via a series of E3 ubiquitin ligases, which, in particular, are required for clearing stalled ribosomes [10]. In addition, amino acids’ oxidation leads to nonspecific protein aggregation and the formation of chaperone foci [10]. Thus, it can be assumed that resistance to 4-NQO requires cells to effectively control protein quality. However, it is surprising that disruption of proteasome activity can lead to increased resistance to some DNA-damaging agents, including 4-NQO [11,12].

To study proteasome-dependent mechanisms of yeast responses to stress, including DNA damage, we previously created mutant strains with decreased expression of the essential proteasome subunits [11,13,14] due to mutation of binding sites (Proteasome Associated Control Element, PACE) for Rpn4, the main regulator of proteasome biogenesis [15,16]. In particular, the YPL strain carries a *pre1–8* PACE mutation in the promoter region of *PRE1*, which encodes an essential structural subunit of the 20S proteolytic proteasome subcomplex [11]. We and other researchers have shown that deregulation of *PRE1* decreases proteasome activity and leads to the phenotypes of impaired proteasome function seen in other strains carrying mutations in the coding region of *PRE1* and other genes encoding proteasome subunits [11,17]. Using a set of these mutants, we showed that disruption of proteasome function leads to an increase in Rpn4-dependent upregulation of key genes of DNA repair systems [11,13]. Thus, we gain insight into how disruption of proteasome function can lead to increased resistance to DNA damage.

Given that UPS is highly conserved, there are a number of evolutionarily conservative relationships between the proteasome and DNA repair systems in yeast and mammals. Additionally, such an evolutionarily conservative link can be provided by the highly conserved ribonucleotide reductase (RNR), an important enzyme that synthesizes dNTPs, which are used for DNA replication and repair [18]. In yeast and mammalian cells, RNR activity is directly or indirectly regulated by UPS to ensure a proper balance between dNTP levels and DNA replication during the cell cycle [19,20] or availability of iron [21], which is an important cofactor for RNRs, DNA polymerases, and other enzymes related to DNA metabolism [22]. According to earlier data, UPS in yeast controls RNR activity indirectly, through the degradation of its protein inhibitors [23,24], whereas in mammals, RNR subunits are themselves UPS substrates [20,25].

The goal of this study is to elucidate a possible proteasome-dependent molecular mechanism of yeast hyper resistance to 4-NQO. Here, we compared the yeast wild-type (WT) strain and the strain with impaired proteasome activity (YPL) using shotgun proteomics. Analysis of the proteomic data, as well as subsequent experiments, showed that the proteasome plays a role in the regulation of nucleotide metabolism through control of RNR activity. Inhibition of the RNR or deletion of its auxiliary proteins reverses the phenotype of yeast hyper resistance to 4-NQO. We showed for the first time that yeast RNR is a substrate of the proteasome, which suggests a common mechanism of RNR regulation in yeast and mammals. Thus, the results obtained in the study of RNR in the yeast model can be translated to mammals.

## 2. Materials and Methods

### 2.1. Yeast Strains

The yeast strains created and used in this work are described in Table 1.

### 2.2. Media, Culture, and Assay Conditions

Yeast cultures were maintained on YPD media. Overnight yeast cultures grown on YPD (yeast extract, peptone, dextrose) media were diluted to OD600 = 0.25 in the fresh YPD media and then grown to log phase (OD600 ≈ 1) at 30 °C. Cells were divided into aliquots, each containing 10^8^ cells, then washed in a PBS buffer and pelleted by centrifugation. The supernatant was quantitatively removed, and cell pellets were stored at −80 °C until proteome analysis by LC-MS/MS.

Cells expressing HA-tagged proteins were grown in selective media without uracil (0.67% (*w*/*v*) yeast nitrogen base without amino acids, 2% glucose, 0.14% Drop-out mix, 0.04% leucine, 0.01% histidine, 0.01% tryptophan, 0.01% adenine, all reagents from Sigma-Aldrich, St. Louis, MO, USA).

### 2.3. LC-MS/MS Data Acquisition

Proteomics data (available from the PRIDE [26] with the dataset identifier PXD014236) were collected earlier as described in [27]. Briefly, cells were lysed in ProteaseMAX Surfactant (Promega, Madison, WI, USA) lysis buffer using ultrasonic homogenization (Bandelin Sonopuls HD2070, Bandelin Electronic, Berlin, Germany). Protein extracts were reduced (10 mM dithiothreitol, 56 °C for 20 min), alkylated (10 mM iodoacetamide, at room temperature for 30 min in the dark), and digested overnight using trypsin (Promega, Madison, WI, USA). LC-MS/MS analysis was performed using an Orbitrap Q Exactive HF mass spectrometer (Thermo Fisher Scientific, San Jose, CA, USA) coupled with an UltiMate 3000 nanoflow LC system (Thermo Fisher Scientific, Germering, Germany) in data-dependent acquisition (DDA), “top 15” mode. A trap column µ-Precolumn C18 PepMap100 (5 µm, 100 Å, 300 µm i.d. × 5 mm) (Thermo Fisher Scientific, Waltham, MA, USA) and an analytical column EASY-Spray PepMap RSLC C18 (2 µm, 100 Å, 75 µm i.d. × 500 mm) (Thermo Fisher Scientific, San Jose, CA, USA) were employed for separations. Mobile phases were as follows: (A) 0.1% formic acid (FA) in water; (B) 95% acetonitrile, 0.1% FA in water. Samples were eluted using the gradient from 5% B to 45% B for 120 min at 270 nL/min flow rate.

### 2.4. LC-MS/MS Data Processing

Raw files were converted to MGF using the MSConvert utility from the ProteoWizard software (ProteoWizard release: 2.1.2575 (TPP v4.5 RAPTURE rev 2, Build 201208012328)) [28]. IdentiPy [29] searches were run against the SwissProt *S. cerevisiae* database (https://www.yeastgenome.org/, accessed on 2 May 2018) using the following parameters: precursor mass tolerance of ±10 ppm, fragment mass tolerance of ±0.01 Da, two allowed missed cleavage sites for trypsin and fixed cysteine carboxyamidomethylation. Search results were then processed and filtered to a 1% false discovery rate (FDR) [30] using Scavager [31].

Each strain was analyzed in three biological replicates, and each was analyzed in three technical replicates. To determine the statistically significant changes in protein abundances, Diffacto software [32] was used. Benjamini–Hochberg correction was employed to account for multiple comparisons [33]. Data visualization was performed using QRePS software (https://github.com/kazakova/Metrics, accessed on 8 December 2022) [34]. Quantification results are provided in Supplemental Material (Figure S1). Differentially regulated features were selected using the following thresholds: |log_2_FC| > 1.2, where FC = YPL/WT; FDR = 0.05. Enrichment analysis was performed separately for down- and upregulated features using Metascape (http://metascape.org, accessed on 17 November 2022) [35].

### 2.5. Real-Time qPCR Analysis

An overnight culture of yeast cells was diluted to OD600 = 0.25 and grown for 4 h at 30 °C with constant shaking. Then, 4-NQO at a concentration of 2 μg/mL was added to the culture medium and incubation was continued for 45 min at 30 °C. No chemicals were added to the control cultures. Cells were pelleted, disrupted with glass beads in a Precellys 24 homogenizer (Bertin Technologies, Montigny-le-bretonneux, France) at 6800 rpm, 3 cycles of 20 s each, in lysis buffer A (Eurogen, Moscow, Russia), and total RNA was isolated using the RNA Solo kit (Eurogen, Moscow, Russia). cDNA was synthesized using RevertAid H Minus Reverse Transcriptase (Thermo Fisher Scientific, Waltham, MA, USA) and the oligo(dT) primer according to the manufacturer’s recommendations. Relative mRNA levels were assessed by real-time qPCR with Eva Green dye (Syntol, Moscow, Russia) using the CFX96 Touch™ Real-Time PCR Detection System (Bio-Rad Laboratories, Hercules, CA, USA). *ACT1* gene was used as a reference. Primary data were processed using the CFX96 Software provided with the instrument and further analyzed in Microsoft Excel (Redmond, WA, USA). The oligonucleotides are listed in Table S1.

### 2.6. Cloning of RNR Genes

The genes encoding all subunits of RNR (*RNR1*, *RNR2*, *RNR3*, and *RNR4*) were cloned into the low-copy yeast shuttle vector YCplac33 [36] under the control of the native promoter and yeast terminator of the *CYC1* gene. For detection purposes, a 3HA epitope was added to the C-terminus of each protein. To avoid nucleotide changes during amplification, the *RNR1* and *RNR3* genes with a 500-nucleotide promoter region were split into two equal parts, and the resulting fragments were amplified with the corresponding primers (Table S1) using KAPA HiFi high-fidelity polymerase (Roche Molecular Systems, Basel, Switzerland). The *RNR2* and *RNR4* genes were each amplified as a single fragment. A short fragment containing the sequence for the epitope 3HA and the *CYC1* terminator was amplified using the previously obtained plasmid pMet4-3HA [14] as a template. The resulting fragments were assembled into corresponding plasmids by recombinational cloning in yeast [37]. Briefly, PCR fragments were mixed equally and transformed into yeast cells by the lithium acetate method [38]. DNA was isolated from the grown colonies and plasmid preparations were tested for correct assembly by PCR. DNA from PCR-positive clones was transformed into competent *E. coli* XL1Blue cells (*recA1 endA1 gyrA96 thi*-1 *hsdR17 supE44 relA1* lac [F’ *proAB lacIqZ*∆M15 Tn10 (Tetr)]) (Eurogen, Moscow, Russia). Plasmid DNA was purified from individual *E. coli* colonies using the GeneJET Plasmid Miniprep Kit (Thermo Fisher Scientific, Waltham, MA, USA) and verified by Sanger sequencing.

### 2.7. Western Blot Analysis of RNR Subunits

The plasmids encoding the RNR genes were transformed into the yeast strain BY4741 (denoted as WT) or the YPL strain. Overnight cultures were grown on selective media without uracil and diluted to OD600 = 0.25. After 2 h of shaking at 30 °C, one half of each culture was treated with 4-NQO (1 μg/mL) for 4 h. The OD600 of each culture was measured and an equal number of cells for each sample was taken. Cell lysates were prepared by alkaline treatment as described in [39]. Lysates were separated on SDS-PAGE (7% for Rnr1 and Rnr3 or 10% for Rnr2 and Rnr4) and transferred to the nitrocellulose membrane. Membranes were blocked with 5% skim milk and incubated with a primary mouse anti-HA antibody (1:2000, Merck, KGaA, Darmstadt, Germany), followed by a secondary anti-mouse antibody conjugated with horseradish peroxidase (HRP) (1:100,000, Jackson Immunoresearch Laboratories, West Grove, PA, USA). The membrane was then incubated in ECL reagents (GE Healthcare Life Sciences, Piscataway, NJ, USA) and developed with Kodak film. Tubulin or Ponceau staining (for 7% PAGE) was used as a loading control. Tubulin was detected using a primary rat monoclonal antibody (1:2000, Abcam, Cambridge, UK) and a secondary horseradish peroxidase-conjugated anti-rat antibody (1:100,000, Jackson ImmunoResearch Laboratories Inc., West Grove, PA, USA). The images obtained were analyzed using ImageJ software [40]. The intensity of proteins of interest was normalized to that of tubulin.

### 2.8. Cycloheximide Chase

Overnight cultures of WT strains expressing Rnr-3HA proteins were diluted to OD600 = 0.25 in a fresh selective medium and continued to grow at 30 °C. After 2 h, polygodial (0.4 μg/mL, a cell-permeabilizing agent [11,41], Sigma-Aldrich, St. Louis, MO, USA) and cycloheximide (CHX, 600 μg/mL, Sigma-Aldrich, St. Louis, MO, USA) were added. Equal samples at time points of 0, 2, 4, and 6 h were taken, the cells were pelleted, and protein samples were prepared for Western blot analysis as described above.

### 2.9. Creation of Yeast Mutants and Inhibitor Plate Assay

Strains with deletions of the *SML1* and *YDJ1* genes were constructed according to the same following scheme. A PCR product containing the *LEU2* gene under the control of its own promoter was amplified using plasmid pGAD (Takara Bio, Kusatsu, Japan) as a template. The amplification primers included 50-nt homology with the flanking sequences of the corresponding target gene (Table S1). The PCR product was transformed into yeast cells by the LiAc/SS carrier DNA/PEG method [38]. The transformants were incubated overnight on liquid selective medium devoid of leucine and then grown on agar plates without leucine for several days at 30 °C. DNA from individual colonies was isolated and the integration of the marker instead of the target gene was confirmed by PCR.

The growth rates of the obtained strains, WTdSML1, WTdYDJ1, YPLdSML1, and YPLdYDJ1, were compared on plates with 4-NQO (0.25 μg/mL) containing hydroxyurea (HU, Sigma-Aldrich, St. Louis, MO, USA) at a concentration of 50 mM. Overnight cultures were diluted to OD600 = 1 and five-fold serial dilutions were prepared for each strain, including control WT and YPL strains. Equal amounts of each dilution (3 μL) were applied to the agar. The plates were incubated at 30 °C for several days with daily photo documentation.

## 3. Results

### 3.1. Proteomic Analysis Indicates Increased Levels of RNR Subunits in the YPL Strain Compared to the WT Strain

**Figure 1:**
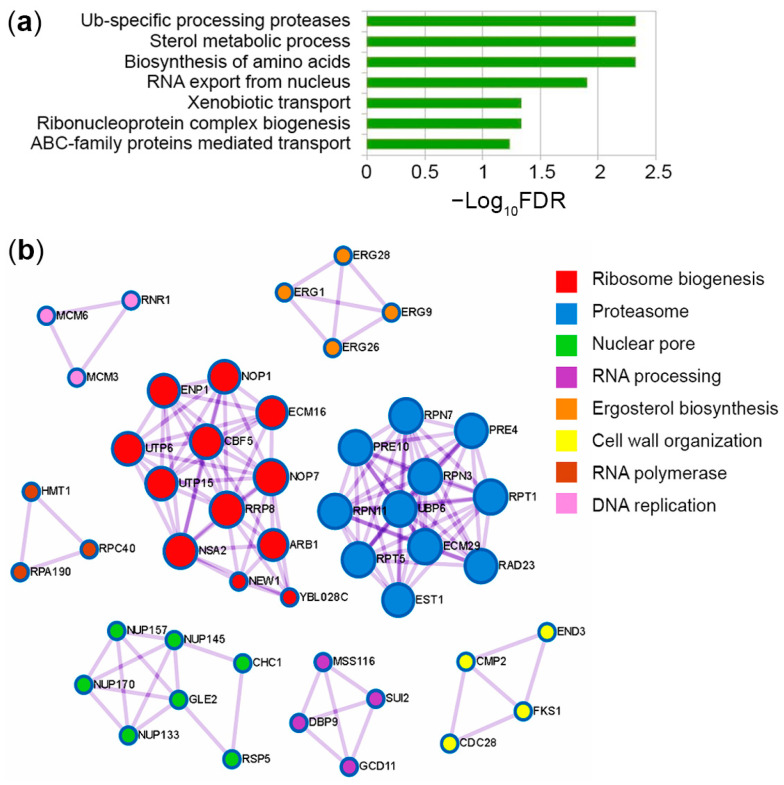
Top enriched terms (**a**) and the molecular complexes (**b**) detected across proteins upregulated in YPL relative to WT strain under normal conditions. The names of the proteins used for the analysis are listed in Table S2. Additional quantitative proteomic data are given in Table S3.

Disruption of proteasome activity negatively affects cellular functions by impairing intracellular protein turnover. Despite this, we and other researchers have previously shown that proteasome mutant strains of yeast are hyper-resistant to several DNA-damaging agents, including 4-NQO [11,12]. Previously, we showed that increased levels of Rpn4-dependent DNA repair genes cause hyper resistance to 4-NQO in proteasome mutants [11,13]. Acquiring resistance should be accompanied by increased levels of proteins managing DNA reparation/replication, as well as degradation of damaged proteins. Therefore, we used LC-MS/MS proteomic analysis in the WT and proteasome mutant strain YPL to search for proteins upregulated in YPL relative to WT that might contribute to hyper resistance to 4-NQO. The raw proteomics data were processed as described in the Methods section to create lists of differentially expressed proteins (DEPs) (Table S2). Then, DEPs were analyzed using the Metascape service [34] and the MCODE algorithm [42] to determine which pathways and protein complexes were enriched in the mutant strain (Figure 1, Table S2). Figure 1a shows the seven significantly enriched terms for proteins whose content was increased in the YPL strain compared with the WT strain. The increased levels of Ub-specific processing proteases suggest that other UPS components (e.g., UBC6, UBP6, ECM29, RSP5 (Table S2)) are activated to compensate for the reduced proteasome activity. The presence of other proteasome subunits among the proteins upregulated in YPL (e.g., PRE4, PRE10, RPT1, RPT5, RPN11) agrees well with our previous results [11,14] and indicates that UPS genes are upregulated in an Rpn4-dependent manner. Increased levels of membrane transporters (e.g., PDR5, PDR15, and SNQ2) in processes such as xenobiotic transport and ABC-family protein-mediated transport also confirm our previous findings [14] and suggest an increased flux of 4-NQO from the YPL strain cells. Proteins (e.g., OSH6, ERG1, ERG9, ERG26, ERG28, ATF2) upregulated in pathways of sterol metabolism, including ergosterol, which is an important component of the yeast cell membrane [43], may also contribute to resistance to 4-NQO by reducing membrane permeability. We also observed enrichment of the ribonucleoprotein complex biogenesis pathway (e.g., GCD11, DBP9, SUI2), which includes proteins involved in ribosome biogenesis as well (e.g., RRP8, NEW1, ARB1, CBF5, ENP1). At first glance, these results look counterintuitive since the stress response is usually accompanied by a decrease in the expression level of ribosomal genes [44]. However, on the other hand, this can be explained by 4-NQO-mediated induction of ribosome quality control [10], which helps to replace ribosomes damaged by oxidation and restore the translation process as much as possible under stress conditions. Increased ribosome biogenesis correlates well with the upregulation of proteins involved in RNA export from the nucleus (e.g., GLE2, NUP133, NUP145, NUP157, NUP170) and enhanced amino acid biosynthesis processes (e.g., SER9, TRP3, TRP4, ILV1, ILV3, MET17) to stimulate translation of proteins, including those that can help to respond to 4-NQO-induced stress. Thus, these data indicate that the YPL strain is more ready to cope with 4-NQO than the WT strain.

In general, the MCODE algorithm identified protein complexes (e.g., proteasome, nuclear pore, proteins involved in ribosome biogenesis) (Figure 1b) relevant to the enriched pathways found by Metascape. The complex cellular response to 4-NQO also includes activation of the DNA damage response [8]. We have previously shown that several DNA repair pathways [11,13] are upregulated in the YPL strain. Accordingly, the MCODE algorithm identified a complex involved in DNA replication and consisting of RNR1, MCM3, and MCM6 (Figure 1b). RNR1 is a large catalytic subunit of the RNR that synthesizes dNTPs, which are used not only for DNA replication but also for repair [18]. Analysis of quantitative data also revealed RNR3 as a significantly upregulated protein in the YPL strain (Table 2). RNR3 is another large subunit of the RNR, a paralog of RNR1 with relatively weak catalytic activity, and is induced by DNA damage [45]. Given the importance of dNTPs biosynthesis for all DNA repair pathways and the fact that the RNR is a target of anticancer therapy, we decided to study the proteasome contribution to the direct regulation of the RNR.

### 3.2. Deregulation of the Proteasome Leads to Enhanced Levels of Large RNR Subunits

**Figure 2:**
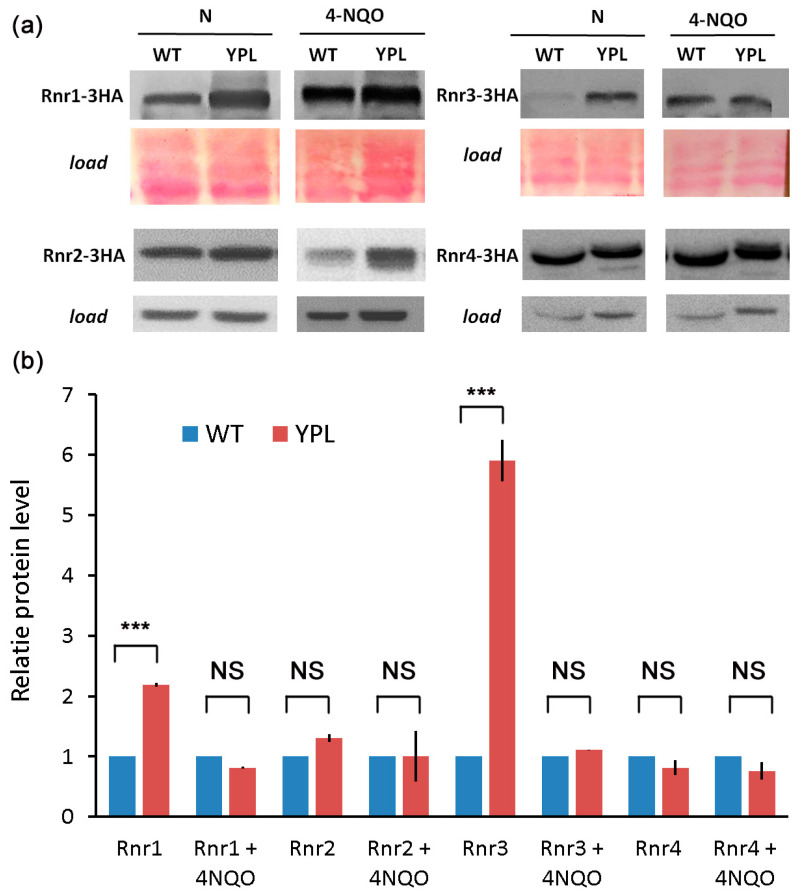
(**a**) Western blot analysis of Rnr-3HA proteins expressed from a low-copy vector in WT and YPL strains confirmed the accumulation of Rnr1 and Rnr3 subunits in the YPL strain compared to the WT strain. Ponceau (for Rnr1,3) or tubulin (for Rnr2,4) staining was used as a loading control. (**b**) Quantification of Western blotting results using ImageJ. The signal of the protein bands in the WT was set to 1. Data represent the mean (*n* = 2) ± SDs. Statistical significance: NS, not significant; *** *p* < 0.001, according to Student’s *t*-test.

Because commercial antibodies are not available for yeast RNR, we labeled each of the four yeast RNR subunits with a 3HA epitope to study their concentration. All subunits (Rnr1-3HA, Rnr2-3HA, Rnr3-3HA, and Rnr4-3HA) were expressed from the same low-copy vector (see “Methods” section). We compared the level of each subunit in the WT strain and the YPL strain under normal growth conditions and after treatment with 4-NQO (Figure 2). According to the results of Western blot analysis, the large subunits of the RNR complex clearly show an increased concentration in the YPL strain under normal conditions, in contrast to the small subunits. This observation agrees well with our proteomic data (Table 2). As for the 4-NQO treatment, the difference between the levels of proteins is not as pronounced (except for the Rnr2 protein), which is consistent with our assumption that the YPL strain is ready to cope with stress.

**Figure 3:**
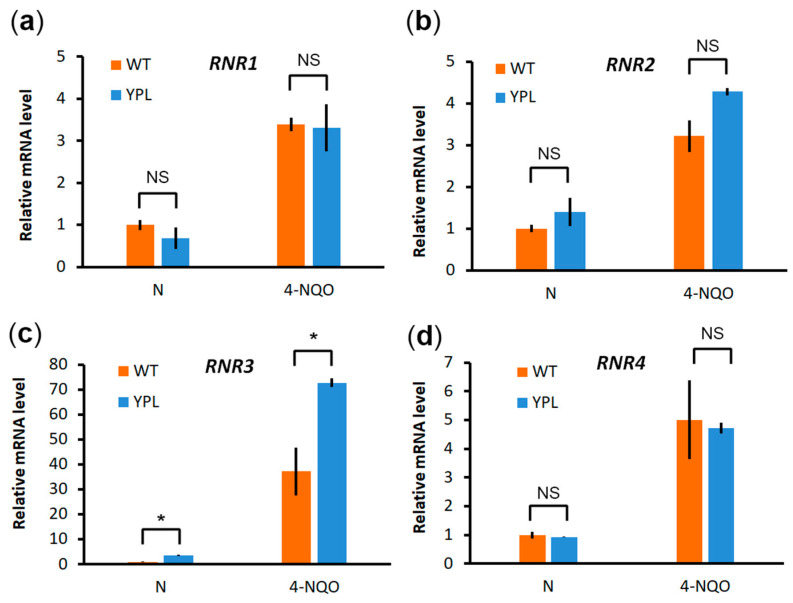
The relative mRNA levels of *RNR1* (**a**), *RNR2* (**b**), and *RNR4* (**d**) genes do not differ significantly in the WT strain from the YPL strain with impaired proteasome activity, whereas for *RNR3* (**c**) they are significantly higher in the YPL strain than in the WT strain. The mRNA levels were measured by RT-PCR under normal conditions or after treatment with 4-NQO at a final concentration of 2 μg/mL for 45 min. *ACT1* was used as a reference. The relative mRNA level in the WT strain under normal conditions was set to 1 for each gene. The plot shows the mean values (*n* = 3) ± SDs. Statistical significance: NS, not significant; * *p* < 0.05, according to Student’s *t*-test.

To confirm that the observed difference between the two strains was indeed due to subunit stabilization rather than to increased gene expression, we compared the mRNA levels of each subunit in both strains, again under normal conditions and under 4-NQO stress (Figure 3). As expected, each is strongly induced by 4-NQO treatment, but there was little difference in gene expression between the two strains, except for *RNR3*, which actually was not part of the RNR complex and apparently has its own function and regulation [46,47,48]. Under stress conditions, upregulation of *RNR3* was again more pronounced in the YPL strain (along with *RNR2*, which tends to have an expression profile similar to *RNR3*); however, we did not observe a strong difference at the protein level. Insignificant differences in mRNA levels of RNR subunits or significant differences in their mRNA levels that did not result in changes at the protein level suggest that RNRs are differentially regulated by altering protein stability.

### 3.3. Rnr1 Is a Proteasome Substrate

**Figure 4:**
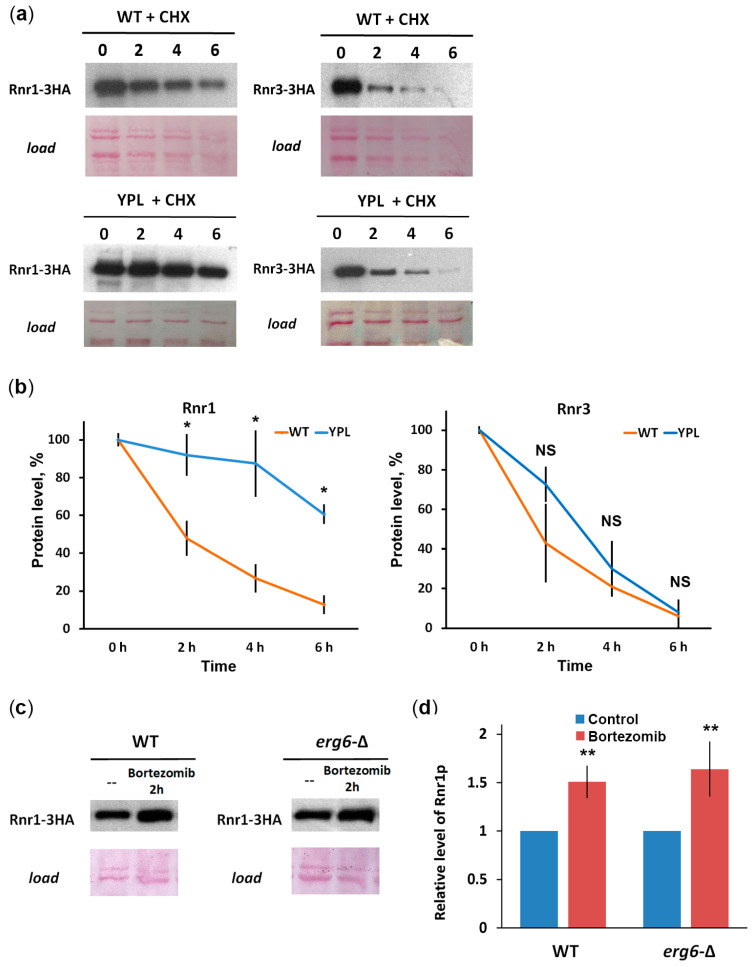
Evaluation of Rnr-3HA protein degradation kinetics by CHX chase. (**a**) Western blot results. Ponceau staining was used as a loading control. Numbers indicate hours of incubation with CHX. (**b**) Quantification of Western blot results with ImageJ after normalization to load density. Plots show mean values (*n* = 2) ± SDs. Statistical significance: NS–nonsignificant; * *p* < 0.05, according to Student’s *t*-test. (**c**) Western blot analysis of Rnr1-3HA protein in WT or *erg6*-∆ strains after treatment with the proteasome inhibitor Bortezomib. Before analysis, yeast transformants in the exponential growth phase were treated with 200 μM Bortezomib for 2 h at 30 °C. Ponceau staining was used as a loading control. (**d**) Quantification of Western blotting results using ImageJ. The signal of the protein bands in WT was set to 1. Data represent the mean (*n* = 2) ± SDs. Statistical significance: NS, not significant; ** 0.01 *p* < 0.001, according to Student’s *t*-test.

To further demonstrate the involvement of the proteasome in the large RNR subunits’ stability, we performed a CHX chase (Figure 4a,b). Indeed, we observed significant stabilization of Rnr1 in the YPL strain (t_1/2_ = 5.35 h) compared with the WT strain (t_1/2_ = 2.39 h), indicating that Rnr1 is a proteasome substrate. In the case of Rnr3, no significant stabilization was observed in the YPL strain (t_1/2_ = 2.58 h) compared to the WT strain (t_1/2_ = 2.25 h). On the one hand, these data suggest that Rnr3 may be degraded in a proteasome-independent manner, for example, by autophagy, as in the case of the mammalian RRM2 subunit [49]. On the other hand, Rnr3 levels can also be affected by changes in gene expression as Figure 3c shows. To provide more evidence that Rnr1 is a proteasome substrate, we used a specific proteasome inhibitor, Bortezomib, and observed Rnr1 accumulation in WT or *erg6*-∆ strains (which has a more permeable membrane for exogenous substances) when treated with Bortezomib (Figure 4c,d). These results strongly suggest that Rnr1 is a substrate of the proteasome.

The proteasome-dependent degradation of Rnr1 in yeast and RRM1 in mammals [25] suggests a common mechanism of RNR regulation and justifies yeast as a model for studying RNR inhibitors.

### 3.4. HU Reverses the 4-NQO Hyper Resistance Phenotype in the YPL Strain

**Figure 5:**
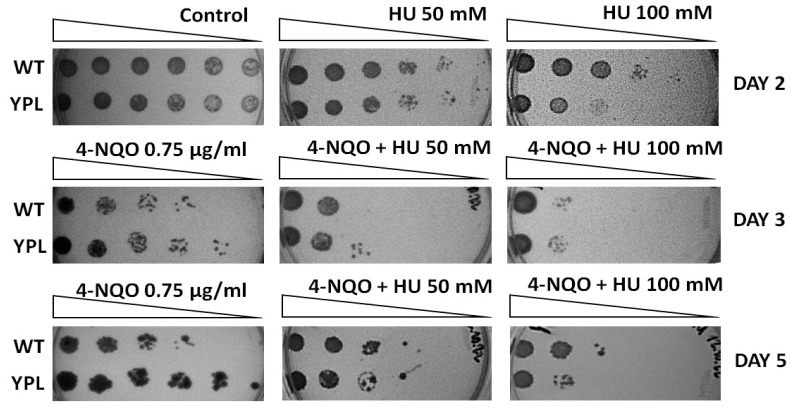
HU inhibits YPL strain growth and reverses its hyper-resistance to 4-NQO. The YPD agar plates with or without (control) additives (HU and/or 4-NQO) were incubated for 5 days at 30 °C.

Next, to assess the contribution of increased subunit content to the viability of the YPL strain under stress, we assayed 4-NQO plates in the presence of the RNR inhibitor HU. Notably, HU is an FDA-approved drug for the treatment of multiple cancers including melanoma, ovarian cancer, and chronic myeloid leukemia [50]. Therefore, this experiment can be considered as a model study of the effect of RNR inhibitors on the phenotype of cancer hyper resistance to DNA-damaging drugs. As previously shown, YPL cells are more viable when chronically exposed to 4-NQO [11,13]. Thus, if increased RNR activity is involved in cell resistance to 4-NQO, its inhibition may cause reduced YPL growth under 4-NQO stress. WT and YPL strains were grown on agar plates with or without the addition of HU in the presence of 4-NQO. The results of the assay (Figure 5) show that the YPL strain no longer exhibits the phenotype of hyper resistance to 4-NQO in the presence of HU. Thus, increased RNR activity contributes to 4-NQO hyper resistance in yeast with impaired proteasome function.

### 3.5. Lack of RNR Regulatory Proteins Sensitizes WT and YPL Strains to HU and 4-NQO

RNR is regulated at different levels, one of which is the level of auxiliary proteins. Two of them, Sml1 [51] and Ydj1 [52], were found to bind subunits of the RNR complex directly and thus regulate the activity of the entire complex. Moreover, the Sml1 protein itself is a well-studied proteasome substrate [23]. Thus, it can be assumed that the accessory proteins may also contribute to the observed resistance of the cells to 4-NQO.

**Figure 6:**
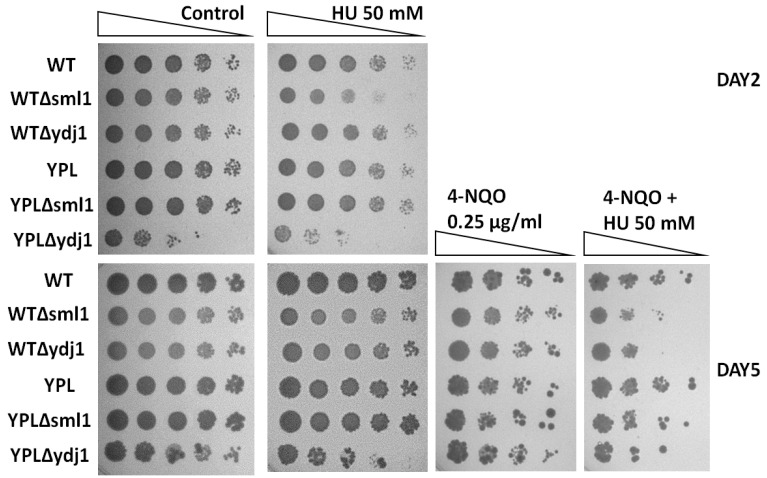
Deletion of the *YDJ1* or *SML1* genes results in inhibition of yeast strain growth in the presence of 4-NQO and HU. YPD agar plates with or without stress agents (control) were incubated for 5 days at 30 °C.

To assess the contribution of RNR regulatory proteins to the observed hyper resistance to 4-NQO under proteasome deregulation, we made deletions of the *SML1* and *YDJ1* genes in the WT and YPL strains. The sensitivity of the mutant strains to 4-NQO and HU was analyzed by plating assay. The results obtained are shown in Figure 6. First of all, we note that in the absence of the *YDJ1* gene encoding the chaperone protein, yeast cells become more sensitive to DNA damage stress, as well as to HU, which agrees well with the results of a previous study [52]. Loss of *YDJ1* impairs the growth of YPL, but not the WT strain under normal conditions and in the presence of HU. These results may indicate an increased role of this chaperone in the regulation of RNR activity and possibly in other cellular processes under proteasome deficiency. In contrast, the absence of *SML1*, which encodes an RNR inhibitor, sensitizes WT, but not YPL cells, to HU. These results may indicate that Sml1 somehow protects the RNR and specifically Rnr1 from degradation by the proteasome. The absence of *YDJ1* or *SML1* increases the sensitivity of WT and YPL strains to 4-NQO in the presence of HU. These data suggest that these proteins may affect RNR activity under stress conditions independently of proteasomal proteolysis.

## 4. Discussion

The *S. cerevisiae* model is a convenient, powerful, and human-relevant model for RNR. RNRs are conserved from yeast to mammals. Both yeast and mammalian RNRs are oligomeric complexes. Yeast RNRs are sensitive to known mammalian RNR inhibitors. Both yeast and mammalian RNRs are tightly regulated at the level of transcription, interaction with protein inhibitors, subcellular localization, degradation by the ubiquitin-proteasome system, and degradation by autophagy. RNR regulation in both yeast and mammals is under the control of DNA damage and DNA replication. Because yeast is fast-growing, easy to cultivate, and cheap to manipulate (including fast and efficient genetic engineering), it can be effectively used in screening procedures to find new RNR inhibitors and to study the molecular details of RNR structure, function, and interactions. We found that proteasome inhibition induces mutant yeast hyper resistance to 4-NQO through stabilization of the catalytic RNR subunit. Inhibition of the RNR or deletion of its regulatory proteins reverses the yeast hyper resistance phenotype to 4-NQO. We showed for the first time that yeast RNR is a substrate of the proteasome.

In order to evaluate the pathways that were upregulated in the YPL strain compared to the WT strain, we performed a proteomic analysis by LC-MS/MS. Consistent with our previous results and those of other authors, we found that upregulated proteins were associated with UPS, membrane transport systems, sterol metabolism, and processes related to ribosome biogenesis and protein translation. We detected subunits of the DNA replication machinery, including RNR, among the upregulated protein complexes. RNR catalyzes the rate-limiting step in dNTP synthesis, which is necessary for DNA repair. Induction of RNR genes under conditions of DNA damage, including 4-NQO treatment, has been shown in both yeast and mammals [46,53,54,55,56]. In our study, we also confirmed a significant increase in the mRNA levels of all four *RNR* genes when treated with 4-NQO (Figure 5).

In addition, Western blot analysis showed that Rnr1 and Rnr3 large subunits tend to accumulate under conditions of decreased proteasome activity as in the YPL strain. According to previous studies, *RNR1* overexpression increases the resistance to 4-NQO of the mutant strain devoid of all genes encoding specialized translesion DNA polymerases [57]. Thus, the accumulation of Rnr1 in the YPL strain may indeed contribute to the hyper-resistance of the YPL strain to 4-NQO. In contrast to the large subunits, we did not observe an accumulation of small subunits Rnr2 or Rnr4 in the YPL strain. This can be explained by the different regulations of large and small subunits at the level of subcellular localization and by accessory proteins. While the large subunits are constantly localized in the cytoplasm, the small subunit subcomplex is mainly located in the nucleus and transported to the cytoplasm during the S-phase or DNA damage [58]. It has also been shown that in cancer cells the small subunit of RNR, RRM2, is degraded by the proteasome in a cell cycle-dependent manner [20]. In contrast, the RRM1 large subunit is subjected to polyubiquitination and proteasomal degradation even in nonsynchronized human cells [25]. Thus, the previous data are consistent with our results and suggest a common mechanism of proteasome involvement in RNR regulation in yeast and human cells.

Unlike mammalian cells, the yeast RNR complex is not tightly regulated by its products (dNTPs) [59]. Instead, the inhibitor protein Sml1 binds the Rnr1 subunit and releases it to form the RNR complex upon DNA damage [51,60]. Its paralog, Dif1, binds the Rnr2-Rnr4 small subunit subcomplex and regulates its subcellular localization along with Wtm1 [24,58]. Additionally, Hug1 and Ydj1 bind to the Rnr2 subunit [52,61]. All of these proteins can affect the stability and availability of RNR subunits for degradation by the proteasome. This assumption well explains our observation that loss of the *SML1* gene makes WT cells sensitive to the HU RNR inhibitor (Figure 6, top panels). At the same time, the effect of *YDJ1* loss, which should protect Rnr2 from inhibition [52], is more pronounced in the YPL background. However, the similar phenotype of strains with *SML1* and *YDJ1* deletions in the presence of 4-NQO and HU regardless of the strain background indicates that the direct regulation of the proteasome subunits does not overlap with their regulation by auxiliary proteins. Thus, the unique diversity of protein regulation levels in yeast does not contradict our general conclusion that the proteasome is directly involved in the regulation of RNR subunits.

Proteasome inhibitors are used to treat several malignancies, such as multiple myeloma [62,63], mantle cell lymphoma [64], acute lymphoblastic leukemia [65], and osteosarcoma [66]. In combinatorial regimens, proteasome inhibitors can be used together with DNA-damaging therapeutic agents [67,68]. Since RNR is a known proteasome substrate in human cells [20,25], it must be stabilized by treatment with proteasome inhibitors. Therefore, our data in yeast as a model organism warn that these combinations may be ineffective due to hyperactivation of the RNR. On the contrary, inhibition of RNR enhances the effectiveness of anticancer therapy [69,70]. Taken together, these data suggest that the combination of proteasome and RNR inhibitors may be more effective in treating cancer. Indeed, the synergistic anti-tumor effect of the combination of 4-hydroxysalicylanilide (targeting RRM2) together with the proteasome inhibitor Bortezomib has been shown in multiple myeloma cell lines, including primary CD138+ cells from Bortezomib-refractory patients [71].

To summarize, we have shown that the ubiquitin–proteasome system regulates yeast RNR activity directly, as has been shown in the case of mammalian RNR. These data further strengthen the thesis that yeast is a relevant model for human RNR studies. Thus, our yeast model can be used in the future to find new good candidates for RNR inhibitors and their combinations with proteasome inhibitors for further testing in human tumor model systems.

## Figures and Tables

**Table 1:** Yeast strains used in the work.

Name of the Strain	Genotype	Source
BY4742	MAT α; *his3*Δ1; *leu2*Δ0; *lys2*Δ0; *ura3*Δ0	Euroscarf, Oberursel, Germany
YPL	BY4742 derivative MAT α; *his3*Δ1; *leu2*Δ0; *lys2*Δ0; *ura3*Δ0, *pre1–8*	[11]
WT△sml1	BY4741 *YML058W*::*LEU2*	This work
WT△ydj1	BY4741 YNL064C::*LEU2*	–//–
YPL△sml1	BY4742 *YML058W*::*LEU2*	–//–
YPL△ydj1	YPL YNL064C::*LEU2*	–//–

**Table 2:** DNA replication-related proteins that are upregulated in the YPL strain compared to the WT strain.

Protein Name	Log_2_FC	−Log_10_FDR ^1^
RNR1	1.52	2.16
RNR3	1.57	3.38
MCM3	1.36	1.74
MCM6	1.23	2.55

^1^ The FDR is Benjamini-Hochberg correction of *p*-values for multiple comparisons.

## Data Availability

Proteomics data are available in PRIDE with dataset identifier PXD014236. All other datasets generated during and/or analyzed during the current study are available from the corresponding author on reasonable request.

## Supplementary Materials

The following supporting information can be downloaded at: https://www.mdpi.com/article/10.3390/jof9030351/s1, Figure S1: Volcano plot for proteins differentially regulated in YPL relative to WT strain; Table S1: Oligonucleotides used in this study; Table S2: List of proteins significantly upregulated in YPL relative to WT, used for the Metascape and MCODE analyses in Figure 1. Table S3: Additional quantitative data for proteins differentially regulated in the YPL strain compared to the WT strain.
