# Supplementary material for: Yeast Ribonucleotide Reductase Is a Direct Target of the Proteasome and Provides Hyper Resistance to the Carcinogen 4-NQO
Source: J Fungi (Basel). 2023 Mar 14;9(3):351. doi: 10.3390/jof9030351 (PMC10057556; doi:10.3390/jof9030351)
Supplement: Supplementary file 1 [file jof-09-00351-s001.zip › Supplementary Data_Figure S1, Table S1.pdf]

Supplementary Data

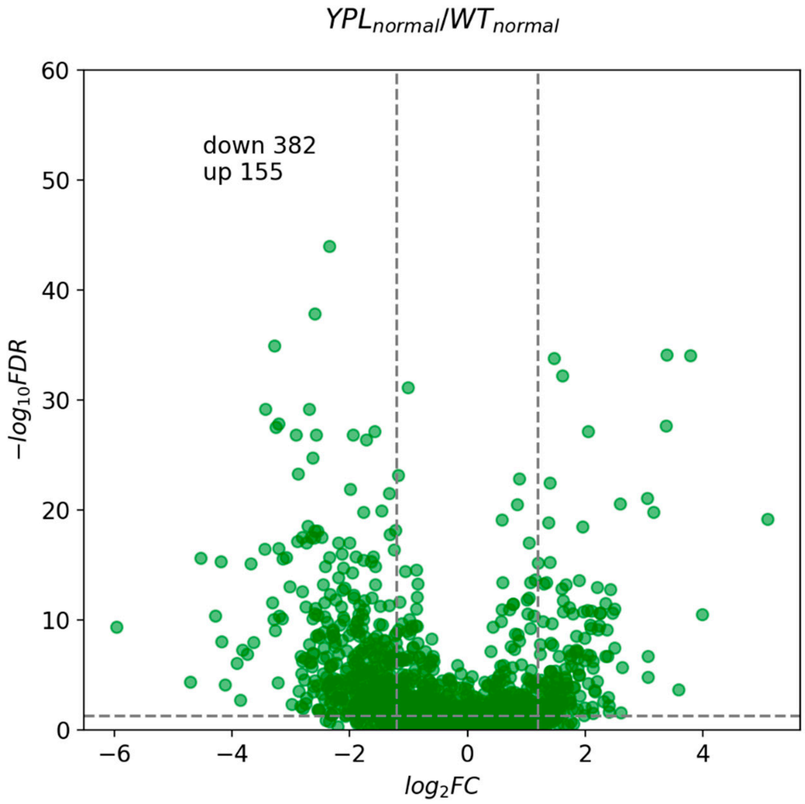

Figure S1. Volcano plot for proteins differentially regulated in YPL relative to WT strain. Differentially regulated proteins are selected using the following thresholds: $|\log_2 FC|$ = 1.2, FC = YPL/WT, FDR = 0.05.

Table S1. Oligonucleotides used in this study

Name	Sequence (5'-->3')	Source
RNR1-ex-top	GTTGCTGATATTTCCAACTTG	This work
RNR1-ex-bot	CTATCTAGAGATGGAATAGTTG	–//–
RNR2-ex-top	TGAAAAAGAGAGGTATGATG	–//–
RNR2-ex-bot	GTCTGGTTTGTTCTTCAAATG	–//–

RNR3-ex-top	TCCAATTTCACATTTGACATG	–//–
RNR3-ex-bot	GGGTACAAAATTCTCTGAAC	–//–
RNR4-ex-top2	CTGGTAACTATGCTTCTATG	–//–
RNR4-ex-bot2	GTTTGGCTTGGTTCTCAAATG	–//–
YCP-RNR1-rec-F	GATAACAATTTCACACAGGAAACAGCTATGACCA TGATTACGCCGCTTGTTTACGCGTTTTATCC	–//–
RNR1-inside-R	GGTCAACATAACGGGCAGTGTTATTGAAAACACGAATC ATAGGAATTAAAC	–//–
RNR1-inside-F	GTTTAATTCCTATGATTCGTGTTTTCAATAACACTGCCC GTTATGTTGACC	–//–
RNR1-3HA-rec-R	CCAGCTGCAGACCCATGACATAACCAATATTAGCCTTTGCA CCCGAACACATTTCACAAGC	–//–
RNR1-3HA-rec-F	GATAACAATTTCACACAGGAAACAGCTATGACCATGATTACGCCGCTTGTTTACGCGTTTTATCC	–//–
tCYC-YCP-rec-R	GAGCTCGGTACGAGAGAAAAATTCGAGCTCGGTACCCGGGGATCCAGCTTGCAAATTAAAGCCTTC	–//–
YCP-RNR2-rec-F	CAATTTCACACAGGAAACAGCTATGACCATGATTACGCCAACTATGCGAAATCCGGAG	–//–
RNR2-3HA-rec-R	CAGCTGCAGACCCATGACATAACCAATATTAGCCTTTGCAAAGTCTTCGTTGAAGGTG	–//–

RNR2-3HA-rec-F	CACCTTCAACGAAGACTTTGCAAAGGCTAATATTGGTTATGTCATGGGTCTGCAGCTG	–//–
YCP-RNR3-rec-F	CAATTTCACACAGGAAACAGCTATGACCATGATTACGCCAAAAGAAAAGAAAAGAAAGTG	–//–
RNR3-inside-R	CTTCCTTACCATGTGTTTTTCTGATATCGACAAAGTCGAAGATATC	–//–
RNR3-inside-F	GATATCTTCGACTTTGTCGATATCAGAAAAACACATGGTAAGGAAG	–//–
RNR3-3HA-rec-R	CAGCTGCAGACCCATGACATAACCAATATTAGCCTTTGCACCGGAACATGACTCACAAG	–//–
RNR3-3HA-rec-F	CTTGTGAGTCATGTTCCGGTGCAAAGGCTAATATTGGTTATGTCATGGGTCTGCAGCTG	–//–
YCP-RNR4-rec-F	GATAACAATTTCACACAGGAAACAGCTATGACCATGATTACGCCGTTCTAGCACACTGAAAGC	–//–
RNR4-3HA-rec-R	CAGCTGCAGACCCATGACATAACCAATATTAGCCTTTGCGAAGTCATCATCAAAGTTAATTTC	–//–
RNR4-3HA-rec-F	GAAATTAACTTTGATGATGACTTCGCAAAGGCTAATATTGGTTATGTCATGGGTCTGCAGCTG	–//–
SML-LEU-F	GATCTTACGGTCTCACTAACCTCTCTTCAACTGCTCAATAATTTCCCGCTAACTGTGGGAATACTCAG	–//–
SML-LEU-R	CAGAACTAGTGGGAAATGGAAAGAGAAAAGAAAAGAGTATGAAAGGAACTGTGCAATTCTTTTTCC	–//–
YDJ-LEU-F	TATCCAAACTGAATTCTACATCTTCCAACAACAATAATAAACGTCCAAAGAACTGTGGGAATACTCAGGTATC	–//–
YDJ-LEU-R	GTATGATGAATAAATGAATCGTGAATAAGTTGATCTTTTTTTATCAAGAAAAGTGCAATTCTTTTTCCTTATC	–//–
